# Breeding of a new reddish-purple fleshed sweetpotato cultivar, ‘Sakurahonoka’, with high yield for processing use

**DOI:** 10.1270/jsbbs.25051

**Published:** 2026-01-20

**Authors:** Takeo Sakaigaichi, Akira Kobayashi, Keisuke Suematsu, Yukari Kawata, Yumi Kai, Rie Kurata

**Affiliations:** 1 Kyushu Okinawa Agricultural Research Center, NARO, Miyakonojo, Miyazaki 885-0091, Japan

**Keywords:** Sakurahonoka, sweetpotato, reddish-purple flesh, pelargonidin, high yield, processing use

## Abstract

We developed a new reddish-purple fleshed sweetpotato (RFSP) cultivar, ‘Sakurahonoka’, with high yield for processing use. Released in 2025, the most distinctive characteristic of Sakurahonoka is its flesh color. The dominant aglycone (anthocyanidin-moiety) of anthocyanin (aglycone) in Sakurahonoka is pelargonidin, which distinctly varied from the purple-fleshed sweetpotato (PFSP) cultivars, whose dominant aglycones were peonidin or cyanidin. Sakurahonoka demonstrated a higher marketable root yield than the two major PFSP cultivars in the Kyushu region of Japan: ‘Ayamurasaki’ and ‘Murasakimasari’. Additionally, Sakurahonoka showed a higher resistance to sweetpotato foot rot disease than Ayamurasaki. Furthermore, the color of Sakurahonoka processed as fried chips and steamed paste was unique and brighter than that of the PFSP cultivars. Food manufacturers have found that Sakurahonoka is suitable as an ingredient in fried chips and boiled-diced sweetpotato. It can be processed into a paste; however, its steamed sweetpotato is fibrous and requires effort to be strained. Thus, Sakurahonoka is an epoch-making RFSP that improves the color variation in sweetpotato-processed foods.

## Introduction

Sweetpotato (*Ipomoea batatas* (L.) Lam.) is a crucial crop in Japan and is used for different purposes, such as baked sweetpotato, distilled liquor, and starch flour (Katayama *et al.* 2017). In particular, the purple fleshed sweetpotato (PFSP), which contains anthocyanins, is recognized as an ingredient in processed foods, distilled liquor, and natural colorants. According to the statistical data (MAFF 2024), ‘Ayamurasaki’ (Yamakawa *et al.* 1997) and ‘Muraskimasari’ (Kumagai *et al.* 2002) are the two major PFSP cultivars in Japan’s Kyushu region. The aglycone (anthocyanidin-moiety) of anthocyanin influences PFSP’s color, and the aglycones in PFSP cultivars are primarily composed of peonidin and cyanidin derivatives (Ishiguro *et al.* 2022, Kurata *et al.* 2024, Oki *et al.* 2002, Tanaka *et al.* 2017, Yoshinaga *et al.* 1999).

Pelargonidin derivatives are considered to be extremely minor anthocyanins in sweetpotato (Truong *et al.* 2010), and few cultivars have been reported to contain pelargonidin (Lee *et al.* 2013). Recently, progress has been made in reddish-purple fleshed sweetpotato (RFSP) breeding containing pelargonidin in Japan. Tanaka *et al.* (2019) showed that the flesh color of sweetpotato clones can be changed to reddish-purple via the dose-dependent selection of the recessive gene in the flavonoid 3ʹ-hydroxylase (*F3ʹH*). Additionally, the Kyushu-Okinawa Agricultural Research Center, National Agriculture and Food Research Organization (NARO/KARC) also released the first RFSP cultivar in Japan named ‘Origin Ruby’ in 2019 (Registration No. 28940).

Despite its novel flesh color, Origin Ruby has not been recommended for cultivation by farmers because its root yield is much lower than that of PFSP cultivars, such as Ayamuraski and Murasakimasari (*Unpublished data*). Recently, resistance to sweetpotato foot rot disease (foot rot disease) (*Diaporthe destruens*) has been considered in cultivars because it caused serious damage in the South Kyushu (Kobayashi 2019, Maeda *et al.* 2022). Cultivars resistant to the disease are required to overcome the yield loss caused by this disease.

Here, we developed a new RFSP cultivar, ‘Sakurahonoka’, whose marketable root yield is higher than that of Ayamurasaki and Murasakimasari. In addition to its high yield, Sakurahonoka is resistant to foot rot disease. Its processed foods, such as fried chips and steamed paste, have a unique bright tone compared to the PFSP cultivars. Therefore, Sakurahonoka is an epoch-making RFSP that improves the color variation in processed sweetpotato.

## Materials and Methods

### Breeding procedures

**Fig. 1.**
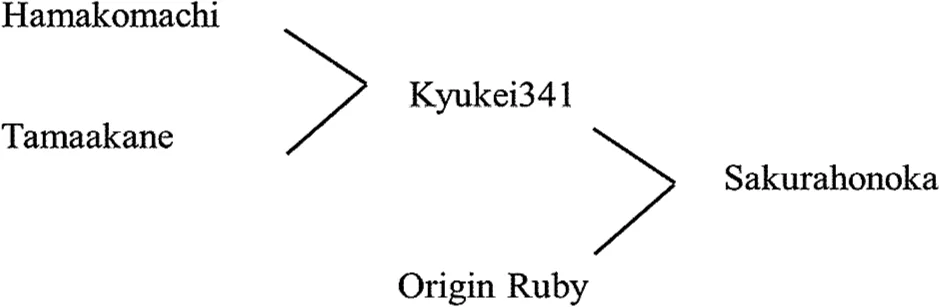
Pedigree of Sakurahonoka.

Fig. 1 shows the pedigree of Sakurahonoka. The female parent ‘Kyukei 341’ is an orange fleshed breeding line with resistance to foot rot disease. The male parent Origin Ruby is a cultivar developed in 2019, and its flesh color is reddish-purple.

Seeds were obtained in 2017 via the artificial pollination of Kyukei 341 and Origin Ruby. The seeds were sown in a greenhouse, and then stem cuttings from each seedling were transplanted to the field of NARO/KARC, Miyakonojo, Miyazaki, Japan (31°45 N, 131°00 E) in 2018. A total of 673 clones were transplanted, and 15 clones with reddish-purple flesh and vigorous root growth were selected at harvest. Line selection and preliminary performance tests were conducted from 2019 to 2021, where the aglycone component of the roots and the marketable root yield (50 g > single root on a fresh matter basis) were also investigated. Through these trials, an elite clone was selected and named ‘Kyukei 376’. A performance yield trial of Kyukei 376 began in 2022, and the local adaptability of Kyukei 376 in Kagoshima Prefecture was investigated. Based on these findings, Kyukei 376 was renamed ‘Kyushu No. 206’, and it underwent further two-year yield trials from 2023 to 2024. Local adaptability in Miyazaki Prefecture had also begun in 2023.

Supplemental Table 1 lists the cultivation conditions for each year. Morphological traits of Sakurahonoka were assessed according to the test guidelines of the International Union for the Protection of New Varieties of Plants (UPOV; Supplemental Table 2). Supplemental Table 3 describes the susceptibility tests for foot rot disease and nematodes. Resistance to foot rot disease was tested in an infested field in Kanoya City, Kagoshima in 2023. Resistance to foot rot disease was ranked into five classes, i.e., strong, slightly strong, medium, slightly weak, and weak, according to Kobayashi *et al.* (2025). Resistance to the southern root-knot nematode (*Meloidogyne incognita*, race SP1) and coffee root-lesion nematode (*Pratylenchus* *coffeae*) was evaluated in the infested fields at Miyakonojo station of KARC/NARO for three years from 2022 to 2024. Furthermore, the nematode resistance was also classified into five ranks from strong to weak, where Ayamurasaki was a check cultivar: “slightly weak” to southern root-knot nematode and “medium” to coffee root-lesion nematode. As a trait related to the sweetness of steamed sweetpotato, brix value was measured according to Kawata *et al.* (2025). The samples were obtained from the yield trials at NARO/KARC in 2022 and 2024.

The suitability of fried chips was assessed for two years (2022–2023) by SHIBUYA FOODS Co., Ltd. (Kochi, Japan). The suitability of boiled-diced sweetpotato and steamed paste was assessed for two years (2022–2023) by Agriprocess Miyazaki Ltd. (Miyazaki, Japan).

Considering all the data, Kyushu No. 206 was recommended for farmers in Kyushu. Thus, it was applied for cultivar registration in July 2025 as Sakurahonoka.

### Evaluations of anthocyanin color value and aglycone composition

The anthocyanin color value (color value), a simple evaluation index used instead of the total anthocyanin content in PFSP studies, was determined according to Sakaigaichi *et al.* (2025). Herein, color value was calculated from the λ max absorbance of 510 or 530 nm and the dilution rate. This is because the λ max absorbance varies among the three aglycone types: 530 nm for peonidin and cyanidin, and 510 nm for pelargonidin. Aglycone composition was determined according to the method described by Kurata *et al.* (2024). Briefly, the supernatant of the anthocyanin extract was hydrolyzed to aglycones (peonidin, cyanidin, and pelargonidin). Subsequently, the percentage of each aglycone was calculated from the ratio of each aglycone content to the total aglycone content by using high-performance liquid chromatography (HPLC).

### Statistical analysis

We performed a two-way analysis of variance (ANOVA) for agronomic traits (marketable root yield, average size of single roots, number of roots per hill, dry matter ratio of roots, and dry matter root yield) and color value among the three cultivars and among the three years. Tukey’s HSD test was used to identify significant differences among the cultivars (*p* < 0.05). All analyses were performed using SPSS ver. 21.0 (IBM, Armonk, NY, USA).

## Results

### Morphological traits

**Fig. 2.**
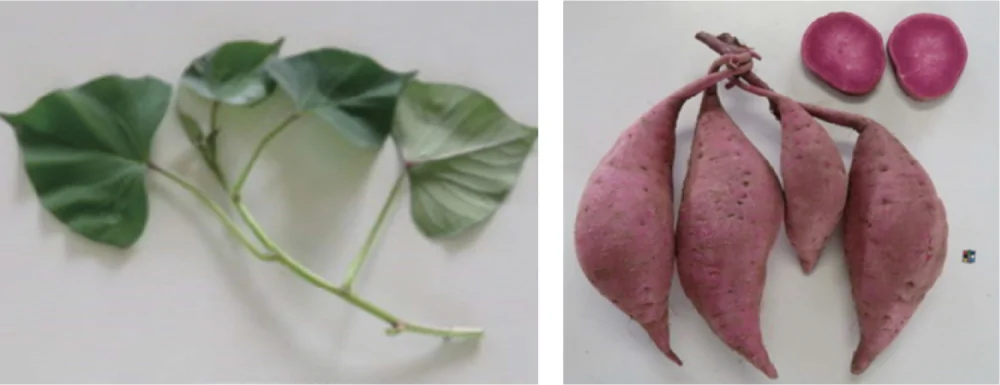
Leaves (left) and storage roots (right) of Sakurahonoka. Photographs of leaves and storage roots were taken on June 6 and September 20, 2024, respevticvely. Photographs were taken using plants grown at the Miyakonojo Station of KARC/NARO.

We assessed the morphological traits of the cultivars according to the test guidelines for UPOV by comparing two major PFSP cultivars, Ayamurasaki and Murasakimasari. Supplemental Table 2 summarized the results. Additionally, Fig. 2 illustrates the photographs of leaves and storage roots of Sakurahonoka. The growth habit of Sakurahonoka was “spreading”. Stem length and diameter of Sakurahonoka were “medium”. The anthocyanin coloration of the internodes in Sakurahonoka was weaker than that in Ayamurasaki and Murasakimasari. The leaves of Sakurahonoka had a “cordate” shape, and this trait was different from Ayamurasaki and Murasakimasari. The color on the upper side of the Sakurahonoka leaves was “green”. The color of the young leaf blade of Sakurahonoka was “light green”. Storage roots of Sakurahonoka had an “elliptic” shape, and the main color of the root skin in Sakurahonoka was “purple red”. The main color of the root flesh was “reddish purple”, and this trait was distinctly different from the PFSP cultivars (Ayamurasaki and Murasakimasari). The intensity of main color of flesh in Sakurahonoka was “medium”.

### Agronomic traits

ANOVA revealed a significant difference (*p* < 0.01) among the cultivars in terms of marketable root yield, average size of single roots, and dry matter ratio of roots (Table 1). The marketable root yield of Sakurahonoka was 352 kg a^–1^ on the three-year average, which was significantly higher than the yields of Ayamurasaki and Murasakimasari (Table 1). The average size of single roots in Sakurahonoka was 218 g on the three-years average (Table 1). The root size of Sakurahonoka was significantly larger than that of Murasakimasari. The number of roots per hill in Sakurahonoka was 4.58 on the three-year average, which was not significantly different from Ayamurasaki and Murasakmasari (Table 1). The dry matter ratio of roots in Sakurahonoka was 31.3% on the three-year average (Table 1). The dry matter ratio was significantly lower than that of Ayamurasaki and Murasakimsari. The dry matter root yield, calculated by multiplying the marketable root yield and the dry matter ratio, was 110 kg a^–1^ in Sakurahonoka and was not significantly different between Ayamurasaki and Murasakimsari (Table 1).

Supplemental Table 3 presents the results of susceptibility tests. Resistance to foot rot disease in Sakurahonoka was “slightly strong”, although Ayamurasaki was “medium”. Sakurahonoka was “strong” to the southern root-knot nematode (race SP1) and “slightly strong” to the coffee root-lesion nematode.

### Flesh color and aglycone composition

**Fig. 3.**
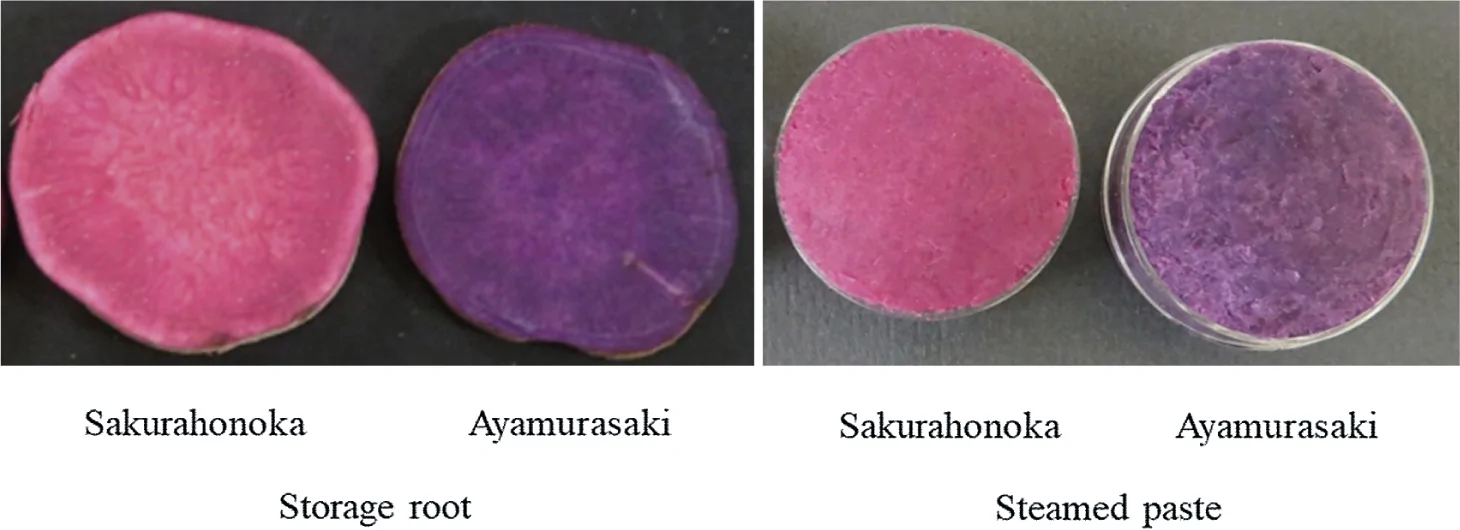
Color of storage root (left) and steamed paste (right) in Sakurahonoka.

Fig. 3 illustrates the flesh color of storage root in Sakurahonoka. Its flesh color was reddish-purple, which was clearly different from that of Ayamurasaki, whose flesh color was purple. The steamed paste of Sakurahonoka was also reddish-purple, which was clearly distinguishable from that of Ayamurasaki (Fig. 3).

**Fig. 4.**
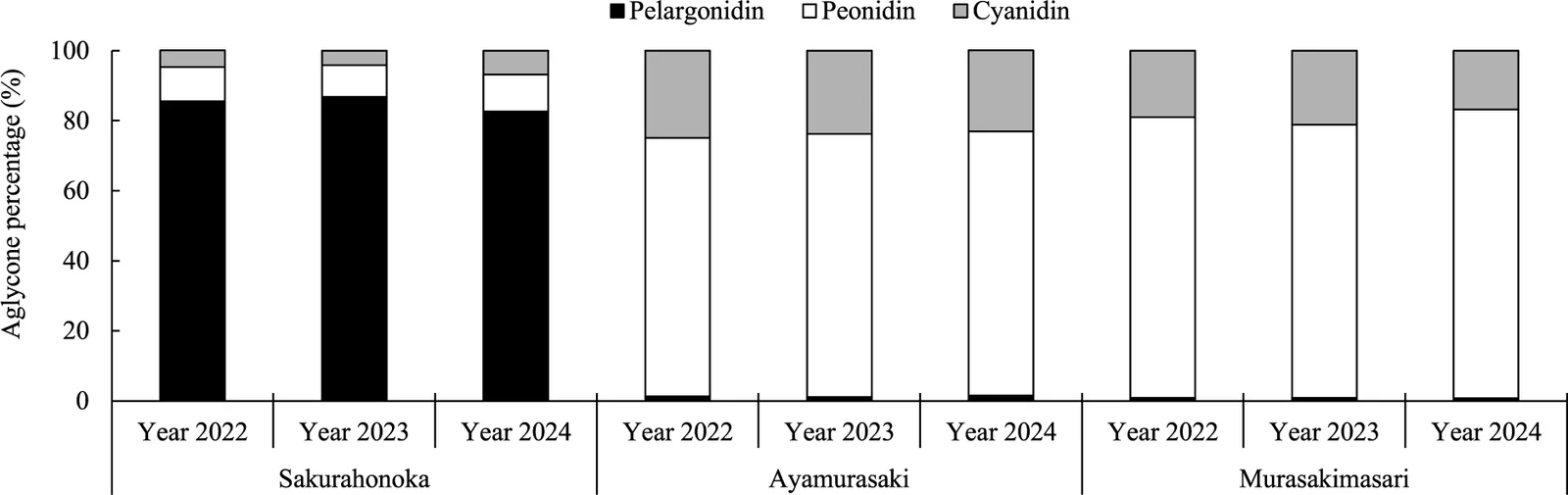
Aglycone percentage of anthocyanins following acid hydrolysis treatment of Sakurahonoka.

The average color value of Sakurahonoka was 2.13 for a three-year average, which was significantly lower than that of Ayamurasaki and Murasakimasari (Table 2). This result indicates that the anthocyanin content was significantly lower in Sakurahonoka than Ayamurasaki and Murasakimasari. Fig. 4 shows the aglycone compositions of Sakurahonoka, Ayamurasaki, and Murasakimasari. The pelargonidin percentage of aglycone was 85.6% in 2022, 86.8% in 2023, and 82.6% in 2024, respectively in Sakurahonoka. However, the pelargonidin percentages of Ayamurasaki and Murasakimasari were less than 2% for the three-year analyses (Fig. 4). In Ayamurasaki and Murasakimasari, peonidin was the most predominant aglycone, followed by cyanidin (Fig. 4).

### Quality of processing use

The brix value of steamed sweetpotato in Sakurahonoka was 14.1% for a two-year average, which was higher than that of Ayamurasaki and Murasakimasari (Supplemental Table 4). The texture of steamed sweetpotato in Sakurahonoka was slightly soggy compared to Ayamurasaki and Murasakimasari.

The suitability of Sakurahonoka for three types of processed products—fried chips, boiled-diced sweetpotato, and steamed pastes—was assessed by classification into five ranks (poor = 1 point, slightly poor = 2 points, medium = 3 points, slightly good = 4 points, and good = 5 points). ‘Tanegashimamurasaki’ was used as the standard PFSP cultivar for the fried chips test. Ayamurasaki is a standard PFSP cultivar used for boiled-diced sweetpotato and steamed paste tests. In these assesments, standard cultivars were classified into “medium” rank having 3 points.

In the fried chips processing, Sakurahonoka was highly evaluated for its exterior compared to Tanegashimamurasaki (Supplemental Table 5). However, flavor, texture, and taste parameters were slightly inferior to those of Tanegashimamurasaki, and the overall evaluation of Sakurahonoka calculated by adding each parameter’s point was almost the same as Tanegashimamurasaki.

In the boiled-diced sweetpotato, Sakurahonoka was highly evaluated in terms of color and hardness parameters compared with Ayamurasaki (Supplemental Table 6). Its taste is almost identical to that of Ayamurasaki. Consequently, Sakurahonoka had a higher overall evaluation score than Ayamurasaki.

In the steamed paste test, Sakurahonoka was also highly assessed in terms of color compared to Ayamurasaki (Supplemental Table 7). Its taste is similar to that of Ayamurasaki. However, Sakurahonoka had a much lower straining score than Ayamurasaki because its steamed paste was fibrous. Thus, its overall evaluation score was slightly lower than that for Ayamurasaki.

## Discussion

Japanese PFSP cultivars such as Ayamurasaki are typically peonidin-dominant, although a few cyanidin-dominant cultivars also exist (Ishiguro *et al.* 2022, Kurata *et al.* 2024, Oki *et al.* 2002, Tanaka *et al.* 2017, Yoshinaga *et al.* 1999). In contrast to peonidin and cyanidin, pelargonidin derivatives are known as extremely minor anthocyanins in PFSP (Truong *et al.* 2010). HPLC analyses in this study clearly showed that Sakurahonoka with reddish-purple flesh was a distinctive pelargonidin dominant clone, and its pelargonidin percentages were stable over 80% for three-years trials (Figs. 3, 4). Sweetpotato is an autohexaploid (2n = 6× = 90) (Shiotani and Kawase 1989), and breeding programs focusing on recessive phenotypes require considerable effort. Reddish-purple flesh is a recessive phenotype in sweetpotato as demonstrated in Tanaka *et al.* (2019). Thus, the successful development of the RFSP cultivar Sakurahonoka is highly valuable for sweetpotato breeding.

No recommendable RFSP cultivars for farmers exist, although Origin Ruby, which has insufficient agronomic traits, has been previously developed. Sakurahonoka is the first-RFSP cultivar with both high yield and resistance to foot rot disease and nematodes. Specifically, the marketable root yield of Sakurahonoka was significantly higher than those of the two major PFSP cultivars, Ayamurasaki and Murasakimasari (Table 1). The foot rot disease resistance in Sakurahonoka was “slightly strong” (Supplemental Table 3), which is in the same classification as a resistant cultivar, ‘Konaishin’ (Kobayashi *et al.* 2025). Sakurahonoka was resistant to southern root-knot and coffee root-lesion nematodes (Supplemental Table 3). Sakurahonoka was concluded to provide sufficient benefits for farmers, considering these agronomic traits.

Ayamurasaki (Yamakawa *et al.* 1997) with high anthocyanin content was an epoch-making cultivar developed in the 1990s, which pioneered new sweetpotato used such as food colorants and ingredients of processed foods. Sakurahonoka has a quite unique flesh color and superior agronomic traits, even when compared to the present major PFSP cultivars in Kyushu. Therefore, Sakurahonoka might be an epoch-making cultivar for sweetpotato breeding in Japan and improves the color variation of sweetpotato processed foods.

In this study, Sakurahonoka was found to be suitable for fried chips and boiled-diced sweetpotato by manufacture’s evaluations (Supplemental Tables 5, 6). Fried chips are an important method of processing sweetpotato. The boiled-diced sweetpotato is used for the topping of breads, cakes, etc. We believe that Sakurahonoka has become popular among the two processing methods. The suitability of steamed paste for sweetpotato processing is a concern because it is widely used as an ingredient in Japanese and Western sweets (e.g., imo-an, and cream). The color of the steamed paste from Sakurahonoka was also superior (Supplemental Table 7). However, Sakurahonoka was fibrous and required labor for straining steamed sweetpotato to make paste (Supplemental Table 7). In the future RFPS breeding, steamed-sweetpotato fibers should be highly valued and fibrous clones should be discarded for the paste use.

## Author Contribution Statement

TS, AK, KS, YK and YK contributed to the breeding of Sakurahonoka. TS and RK analyzed the anthocyanin. TS drafted the manuscript, and all authors approved the final manuscript.

## Supplementary Material

Supplemental Tables

## Figures and Tables

**Table 1.** Yield performance of Sakurahonoka

Trait	Cultiver	Year			Mean^*b*^	F-value^*c*^		
		2022	2023	2024		Cultiver (C)	Year (Y)	C × Y
Marketable root yeild^*a*^ kg a^–1^	Sakurahonoka	357	320	379	352 a	19.53**	8.52*	0.21
	Ayamurasaki	300	237	319	285 b			
	Murasakimasari	304	257	312	291 b			
Average size of single root g	Sakurahonoka	244	189	220	218 a	35.80**	98.03**	0.75
	Ayamurasaki	234	164	170	189 ab			
	Murasakimasari	204	128	157	163 b			
Number of roots per hill	Sakurahonoka	4.00	4.82	4.92	4.58 a	5.46	34.25**	3.20
	Ayamurasaki	3.50	4.20	5.29	4.33 a			
	Murasakimasari	4.10	5.50	5.57	5.06 a			
Dry matter ratio of roots %	Sakurahonoka	30.7	31.4	31.8	31.3 c	87.92**	3.12	2.64
	Ayamurasaki	33.9	34.5	32.2	33.5 b			
	Murasakimasari	37.5	37.6	35.9	37.0 a			
Dry matter root yeild kg a^–1^	Sakurahonoka	110	101	120	110 a	5.67	4.15	0.24
	Ayamurasaki	102	82	103	95 a			
	Murasakimasari	115	97	112	108 a			

^*a*^ Marketable root yield indicates fresh matter root yield with indivudual weights >50 g. ^*b*^ Values followed by the same alphabet are not significantly different for each traits by Tukey’s HSD test (*p* < 0.05). ^*c*^ * *p* < 0.05, ** *p* < 0.01 by two-way ANOVA.

**Table 2.** Anthocyanin color value of Sakurahonoka

Trait	Cultiver	Year			Mean^*a*^	F-value^*b*^		
		2022	2023	2024		Cultiver (C)	Year (Y)	C × Y
Anthocyanin color value of roots	Sakurahonoka	2.70	2.20	1.49	2.13 b	241.11**	20.99**	4.74*
	Ayamurasaki	6.10	5.40	3.83	5.11 a			
	Murasakimasari	6.20	4.80	4.46	5.15 a			

^*a*^ Values followed by the same alphabet are not significantly different for each traits by Tukey’s HSD test (*p* < 0.05). ^*b*^ * *p* < 0.05, ** *p* < 0.01 by two-way ANOVA.
