# Supplementary material for: Breeding of a new reddish-purple fleshed sweetpotato cultivar, ‘Sakurahonoka’, with high yield for processing use
Source: Breed Sci. 2026 Jan 20;76(2):190–5. doi: 10.1270/jsbbs.25051 (PMC13494325; doi:10.1270/jsbbs.25051)
Supplement: Supplementary file 1 [file 76_190_s1.pdf]

doi: 10.1270/jsbbs.25051

**Supplemental Table 1.** Cultivation conditions of the yield trials in 2022–2024.

| Year | Date | | No. of plots | No. of hills per plot | Planting density cm | Mulch film | Fertilization kg $a^{-1}$ | | | |
|---|---|---|---|---|---|---|---|---|---|---|
| | Transplant | Harvest | | | | | N | $P_2O_5$ | $K_2O$ | Manure |
| 2022 | 22-Apr | 27-Sep | 3 | 48 | 75×35 | Black | 0.48 | 0.72 | 1.20 | 100 |
| 2023 | 17-May | 26-Sep | 3 | 48 | 75×35 | Black | 0.48 | 0.72 | 1.20 | 100 |
| 2024 | 25-Apr | 27-Sep | 3 | 48 | 75×35 | Black | 0.48 | 0.72 | 1.20 | 100 |

**Supplemental Table 2.** Morphological traits assessed according to the test guidelines of UPOV.

| No. | Traits | Cultivar | | |
|---|---|---|---|---|
| | | **Sakurahonoka** | **Ayamurasaki** | **Murasakimasari** |
| 1 | Plant: growth habit | spreading | spreading | spreading |
| 2 | Stem: length of primary shoots | medium | medium | short to medium |
| 4 | Stem: diameter of internode | medium | medium | medium |
| 5 | Stem: anthocyanin coloration of internode | weak | medium | medium |
| 9 | Leaf blade: number of lobes | absent | 3 | 3 |
| 10 | Leaf blade: shape | cordate | - | - |
| 12 | Leaf blade: color on upper side | green | green | green |
| 16 | Young leaf blade: main color on upperside | light green | purplish brown | light green |
| 19 | Storage root: shape | elliptic | elliptic | ovate |
| 22 | Storage root: main color of skin | purple red | red | red |
| 24 | Storage root: main color of flesh | reddish purple[a] | purple | purple |
| 25 | Storage root: intensity of main color of flesh | medium | dark | dark |

[a]Underlined 'reddish purple' (No.24 in Sakurahonoka) is a flesh color that dose not exsit in the current UPOV category.

**Supplemental Table 3.** Resistance to sweetpotato foot rot disease and nematodes.

| Trait | Cultivar | Ressisatance |
|---|---|---|
| Sweetpotato foot rot disease[a] | Sakurahonoka | Slightly strong |
| | Ayamrasaki | Medium |
| Southern root-knot nematode (race SP1) [b] | Sakurahonoka | Strong |
| | Ayamrasaki | Slightly weak |
| | Murasakimasari | Slightly strong |
| Coffee root-lesion nematode[c] | Sakurahonoka | Slightly strong |
| | Ayamrasaki | Medium |
| | Murasakimasari | Strong |

[a]Resistance was tested in an infested field in Kanoya City, Kagoshima, in 2023.

[b]Resistance was assessed in the infested field at the Miyakonoyo Station of KARC/NARO from 2022 to 2024.

[c]Resistance was assessed in the infested field at the Miyakonoyo Station of KARC/NARO from 2022 to 2024.

**Supplemental Table 4.** Brix value of steamed sweetpotato.

| Trait | Cultiver | Year | | Mean |
|---|---|---|---|---|
| | | 2022 | 2024 | |
| Brix value % | Sakurahonoka | 14.1 | 14.0 | 14.1 |
| | Ayamurasaki | 10.9 | 7.6 | 9.3 |
| | Murasakimasari | 13.6 | 9.6 | 11.6 |

The samples were obtained from the yield trials at NARO/KARC.

**Supplemental Table 5.** Evaluation of the suitability of fried chips ranked from 1 (poor) to 5 (good).

| Parameter | Cultivar | Year | | |
|---|---|---|---|---|
| | | 2022 | 2023 | Mean |
| Exterior | Sakurahonoka | 4.0 | 3.7 | 3.9 |
| | Tanegashimamurasaki[a] | 3.0 | 3.0 | 3.0 |
| Flavor | Sakurahonoka | 2.7 | 2.7 | 2.7 |
| | Tanegashimamurasaki | 3.0 | 3.0 | 3.0 |
| Texture | Sakurahonoka | 2.5 | 2.8 | 2.7 |
| | Tanegashimamurasaki | 3.0 | 3.0 | 3.0 |
| Taste | Sakurahonoka | 2.7 | 2.6 | 2.7 |
| | Tanegashimamurasaki | 3.0 | 3.0 | 3.0 |
| Overall evaluation | Sakurahonoka | 11.9 | 11.8 | 11.9 |
| | Tanegashimamurasaki | 12.0 | 12.0 | 12.0 |

[a]Standard cultivar of the purple sweetpotato suitable for fried chips

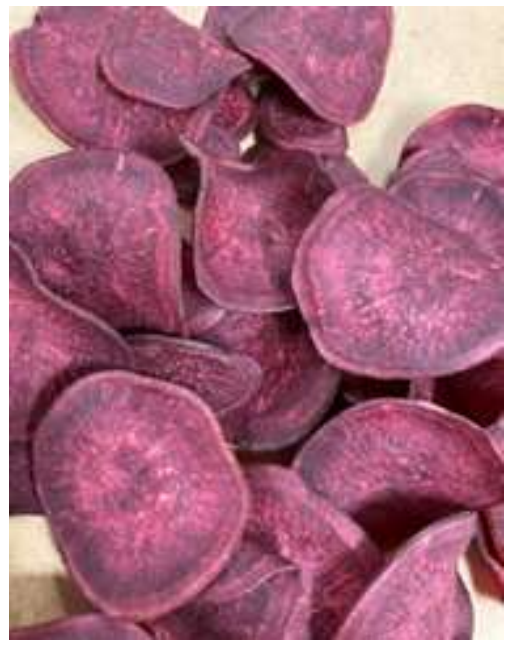

Fried chips: Sakurahonoka

**Supplemental Table 6.** Evaluation of the suitability of boiled-diced sweetpotato ranked from 1 (poor) to 5 (good).

| Parameter | Cultivar | Year | | Mean |
|---|---|---|---|---|
| | | 2022 | 2023 | |
| Color | Sakurahonoka | 4.0 | 5.0 | 4.5 |
| | Ayamurasaki[a] | 3.0 | 3.0 | 3.0 |
| Hardness | Sakurahonoka | 4.0 | 5.0 | 4.5 |
| | Ayamurasaki | 3.0 | 3.0 | 3.0 |
| Taste | Sakurahonoka | 2.0 | 4.0 | 3.0 |
| | Ayamurasaki | 3.0 | 3.0 | 3.0 |
| Overall evaluation | Sakurahonoka | 10.0 | 14.0 | 12.0 |
| | Ayamurasaki | 9.0 | 9.0 | 9.0 |

[a]Standard cultivar of the purple sweetpotato

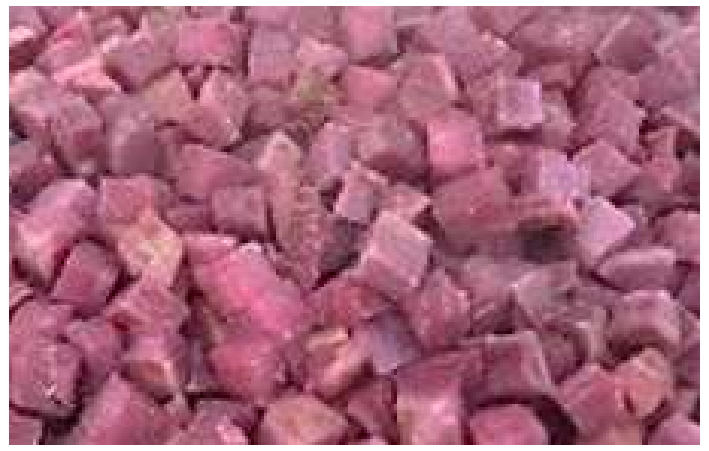

boiled-diced sweetpotato: Sakurahonoka

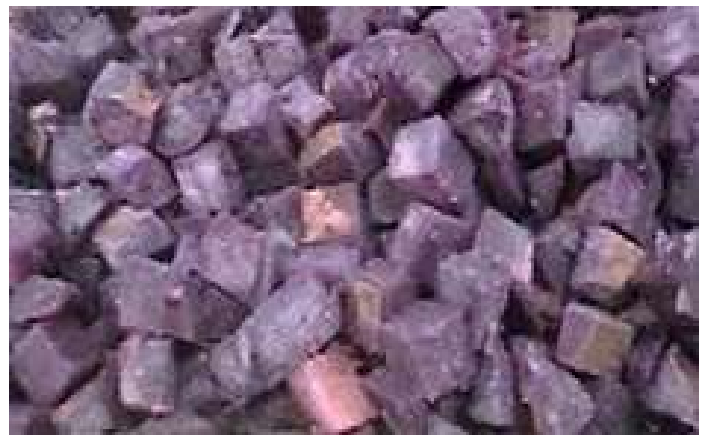

boiled-diced sweetpotato: Ayamurasaki

**Supplemental Table 7.** Evaluation of the suitability of steamed paste ranked from 1 (poor) to 5 (good).

| Parameter | Cultivar | Year 2022 | Year 2023 | Mean |
|---|---|---|---|---|
| Color | Sakurahonoka | 4.0 | 5.0 | 4.5 |
| | Ayamurasaki[a] | 3.0 | 3.0 | 3.0 |
| Taste | Sakurahonoka | 3.0 | 3.0 | 3.0 |
| | Ayamurasaki | 3.0 | 3.0 | 3.0 |
| Straining | Sakurahonoka | 1.0 | 1.0 | 1.0 |
| | Ayamurasaki | 3.0 | 3.0 | 3.0 |
| Overall evaluation | Sakurahonoka | 8.0 | 9.0 | 8.5 |
| | Ayamurasaki | 9.0 | 9.0 | 9.0 |

[a]Standard cultivar of the purple sweetpotato

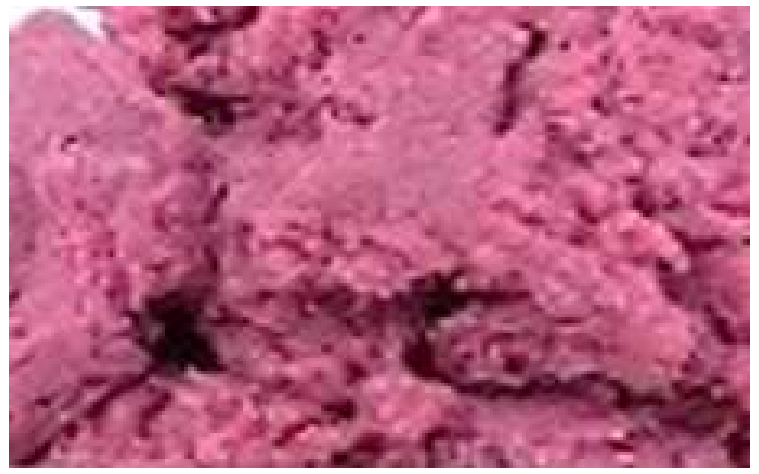

Steamed paste: Sakurahonoka

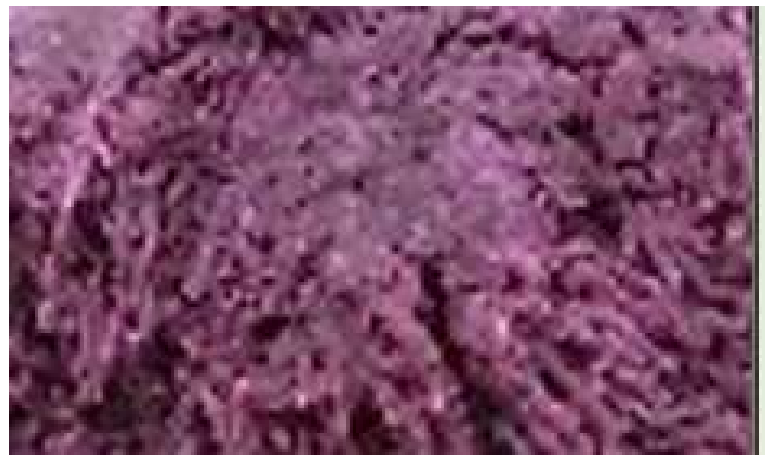

Steamed paste: Ayamurasaki
